# Dental Jurisprudence.—Infection

**Published:** 1895-12

**Authors:** H. R. Wiley

**Affiliations:** San Francisco.


ARTICLE VIII.

### DENTAL JURISPRUDENCE.—INFECTION.

BY H. R. WILEY, A. B., SAN FRANCISCO.

The records of Dental Jurisprudence disclose the fact that less litigation has arisen on account of the infection of disease, through the use of dental instruments, than the investigator would naturally expect to find.

Supposing that the average number of thoughtless or careless individuals may be found among the members of the dental profession, it is but reasonable to presume that many serious cases of poisoning have been caused by the use of unclean instruments. Though this view of the matter may not be sustained by our courts, yet the annals of the profession establish it to a moral certainty.

Dr. Rehfuss cites a number of cases in point, collected from the *Dental Mirror* and other sources. The majority of the cases referred to were of syphilitic poisoning, presumed to have been caused by slight abrasions produced by the slipping of the instruments during dental operations.

Though each of these cases seemed traceable directly to the operations of a dentist, and no other reasonable theory appeared by which to explain the inoculation, yet, it is possible that in more than one case the dentist was unjustly blamed.

When we consider the fact, that it is held, upon good authority that infection of such disease may occur through the simple and evanescent agency of a kiss, or through contact of the inner surface of the lip wtth the poisoned surface of a drinking cup, we cannot fail to realize that serious difficulty must attend the efforts of the investigator who would arrive at any degree of certainty on the point under consideration.

An appreciation of the difficulty referred to undoubtedly has deterred some individuals from prosecuting suits for damages against dentists, through whose supposed criminal carelessness they believed themselves to have suffered.

The danger of infection, through the medium of surgical instruments, is now fully understood by the intelligent practitioner, and has been ably met by the introduction of antiseptics. A thorough disinfection of surgical instruments, both before and after an operation, is now recognized as an essential principle in surgery, and is applicable to its every branch.

The general recognition of this principle by the profession involves an increase of responsibility on the part of the dentist, and therein lies its legal significance.

The law demands of the dentist that he shall, in all matters involving serious danger to his patient, reduce that danger to a minimum by the use of such means as are recognized and advocated by the authorities of his profession, or such other means as may be proven to be equivalent thereto.

If the dentist does not possess reasonable knowledge of the proper means and methods to employ in a given case, he is chargeable with ignorance; if he possesses the knowledge of the means, but neglects to use them skillfully, he is chargeable with negligence, and under either condition is liable for any injury proven to be the direct result therefrom.

The dentist's safe course lies in acquainting himself with the best means and methods in present use in his profession for preventing infection, and in the employment, uniformly, of those means and methods in his practice.

In case of suit for damages for injuries alleged to have been caused by the use of unclean instruments, in the absence of absolute proof connecting the injury with the operation, the dentist will find a strong defense in being able to prove that he has followed the course above outlined.—*Pacific Stomatological Gazette.*

---

